# Urgent need to take action on reducing postoperative respiratory complications

**DOI:** 10.1016/j.lanwpc.2021.100136

**Published:** 2021-04-12

**Authors:** Lan My Le, Nathorn Chaiyakunapruk

**Affiliations:** a Swiss Tropical and Public Health Institute [Swiss TPH], Basel, Switzerland; b University of Basel, Basel, Switzerland; c Department of Pharmacotherapy, University of Utah, Salt Lake City, UT, USA

**Keywords:** Postoperative respiratory complications, Economic burden, Re-hospitalization, Vietnam

Post-surgical complications are common in general surgery patients and contribute to increases in mortality, length of stay, and need for an increased level of care at discharge [1,2]. Postoperative respiratory complications (PRCs), the second most common serious morbidity after cardiovascular events [3], are broadly defined as conditions affecting the respiratory tract that can adversely influence clinical course of the patient after surgery. PRCs occur commonly with incidence estimates of 3 -7.9% in general surgery, have major adverse effects on patients, and are difficult to predict.

In addition to being an important clinical problem, post-operative complications in general, and PRCs pose heavy economic burdens. Several studies, mostly in high income countries, confirmed that patients with one or more complications after surgery required longer length of stay or rehospitalization which resulted in higher hospitalization costs [4], [5], [6], [7]. In the face of that, evidence on economic burden of complications after surgery in low-middle-income countries (LMICs) are limited.

In *The Lancet Regional Health – Western Pacific*, My Hanh Bui and colleagues quantify the impact of PRCs on costs, re-hospitalization, outpatient visits, and length of hospital stay (LOS) within 30 days after surgery from the healthcare sector perspective in Vietnam [8]. The data of 1 241 893 surgical patients were extracted from electronic payment portal database of Vietnam Social Insurance from 1^st^ Jan 2017 to 30^th^ Sept 2018. Of these patients, 20 051 patients (16%) had PRCs which were grouped into 10 categories. The study applied the propensity score matching method with a matched ratio of 1:1 and identified 13 006 patients with and without PRCs. An important finding was that the patients with PRCs had a higher risk of re-hospitalization (OR: 3.49; 95% CI: 3.55 – 3.64) and outpatient visit (OR: 1.39, 95% CI: 1.34 – 1.45) compared to patients without PRCs. Most types of costs were significantly higher for patients with PRCs. The incremental cost associated with PRCs occurring within 30 days after surgery was $1053.3 (95% CI: 940.7 – 1,165.8) in which the largest contribution was the cost of re-hospitalization. Their study estimated that annual incremental cost due to PRCs was $6.02 and $13.87 million for indexed treatment and the 30-day cost, respectively. The study demonstrates high economic evidence associated with PRCs for Vietnam, as an example of LMICs, justifying the urgent need to lower the incidence of preventable post-surgical complications.

The surgical safety checklist (SSC) was introduced in 2009 by WHO with an aim to reduce the complication rates during the surgical process [9]. This initiative enables surgeons, anesthesiologists, nurses, and other health care staff to routinely check information at three critical stages of surgery to reduce operative errors and improve outcomes. SSC has been trialed in hospitals worldwide to validate its feasibility and effectiveness. Several studies confirmed that the adoption of SSC could reduce morbidity and mortality due to adverse events after surgery in the poor-resource settings [10], [11], [12]. The benefits somehow are more critical in developing countries compared to developed ones. For example, in settings with limited financial and human resources where surgeons and operative teams have to perform several surgeries continuously, a checklist would be useful to avoid mistakes and ensure patient safety. Despite the evidence on benefits of SSC, it cannot be assumed that the introduction of SSC will immediately improve the operative processes. Hence, training and practice for staff is needed to ensure successful implementation. As different countries and settings have different social norms, it is necessary to modify SSC for cultural variations to optimize the feasibility and usefulness of SSC and improve its compliance in LMICs.

Although surgical safety has been addressed by the adoption of checklists, intervention beyond the operating room has been limited for developing countries. Transitional care refers to the coordination and continuity of services for the movement either between levels of health care or from one healthcare setting to another or to home [13]. In urban areas in high income countries, transitional care, especially from hospital to home after surgery has proven to prevent post-surgical complications [14,15]. However, its implementation received little attention in low-income settings [16]. A considerable infrastructure is required, as is the coordination of a multidisciplinary team including surgeon, nurse, pharmacist, etc. Hence, a discussion synchronization on the hospital care system and services as well as allocation of health resources may be necessary. Moreover, awareness enhancement on surgical recovery, especially on post-surgical complications for all persons involved in surgery and post-surgical recovery is critical.

Post-operative complications, particularly PRCs substantially increase health care costs which results in economic burden to the health care system and patients in high income countries [4], [5], [6], [7]. Bui My Hanh, and colleagues take an important step forward for understanding clinical and economic outcomes of PRCs in Vietnam. The authors suggest implementing evidence-based practices and monitoring systems to lower the incidence of PRCs which in turn reduce costs. Interventions to improve surgical safety through checklists may hold many possibilities of future application. Advancing the health care system to provide better transitional care for surgical patients may be challenging but may also bring an exciting opportunity to further drive down adverse outcomes within surgery, particularly in developing settings. It would also be important to determine whether these preventive strategies in pre- or post-surgery could decrease the number of re-admission and subsequent costs. A comprehensive assessment of these potential interventions including clinical effectiveness, cost-effectiveness, feasibility, and associated budget implications should be performed to support evidence-informed policy decision making.

## Declaration of Competing Interest

The authors declare no competing interests.
